# Etymologia: Fluoroquinolone

**DOI:** 10.3201/eid2305.ET2305

**Published:** 2017-05

**Authors:** Ronne Henry

**Keywords:** fluoroquinolone, quinolone, nalidixic acid, norfloxacin, ciprofloxacin, drugs, antimicrobial resistance, fluorine, carbon, Bayer, Kyorin

## Fluoroquinolone [floorʺo-kwinʹo-lōn]

The first quinolone (*quinol*[ine] + -*one* [compound related to ketone]), nalidixic acid (Figure), was isolated as a byproduct of chloroquine (see “quinine”) synthesis and was introduced in 1962 to treat urinary tract infections. In 1980, researchers at the Kyorin Pharmaceutical Company showed that the addition of a fluorine atom to the quinolone ring resulted in an antibiotic with broader antimicrobial activity, which was named norfloxacin, the first fluoroquinolone. In 1983, Bayer published data that showed adding a single carbon atom to norfloxacin—what would become ciprofloxacin—further increased activity. Fluoroquinolones are today among the most frequently used antimicrobial drugs to treat infections in humans and animals.

**Figure:**
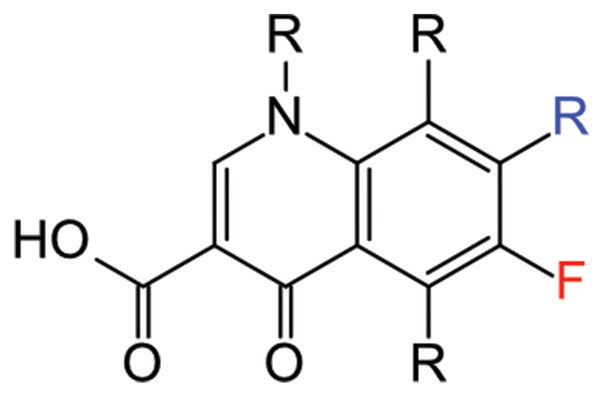
By Reubot, Public domain, Wikimedia Commons, https://commons.wikimedia.org/w/index.php?curid=14746558
